# The financing sustainability of long-term care insurance: an example from Nanning, China

**DOI:** 10.3389/fpubh.2025.1454037

**Published:** 2025-04-22

**Authors:** Butong Chen, Xuhao Zhu, Huiyu Wang, Lijie Zong, Junqi Feng, Sijing Tu, Qianqiang Wang

**Affiliations:** ^1^College of Humanities and Social Sciences, Guangxi Medical University, Nanning, China; ^2^Department of Medical Insurance, Yueyang Central Hospital, Yueyang, China; ^3^Wuhan Wuchang Hospital, Wuhan, China; ^4^School of Public Health and Management, Guangxi University of Chinese Medicine, Nanning, China; ^5^School of Public Health, Hangzhou Normal University, Hangzhou, China

**Keywords:** long-term care insurance, financing mechanism, sustainable operation, aged care, disability care

## Abstract

**Objective:**

With accelerated population aging in China, older adult services and long-term care for people with disabilities are becoming serious problems. Currently, mobilizing and allocating social resources, establishing reasonable financing mechanisms to address the growing demand for long-term care for those with disabilities, and ensuring the sustainable operation of long-term care insurance financing mechanism are vital.

**Methods:**

Taking Nanning, China, as an example, based on the Nanning Statistical Yearbook and relevant policy documents of government departments such as the medical care and security department, we use the PADIS-INT model and the International Labour Organization financing model(ILO Model) to estimate the contribution levels of Nanning’s residents’ long-term care insurance for the years of 2025, 2030, and 2035, and analyze the feasibility of the government’s and rural and urban residents’ individual contribution burden.

**Results:**

The average annual contribution amount of long-term care insurance for urban and rural residents will increase from 108.80 yuan in 2025 to 202.71 yuan in 2035. According to the sharing method, in which the government and individuals each bear 50% of the financing responsibility for long-term care insurance, the proportion of the government’s financial responsibility for the cost of long-term care insurance for urban and rural residents to the current year”s financial income will be 0.34, 0.42, and 0.50% in 2025, 2030, and 2035, respectively, and the amount of money that can be used by individual urban and rural residents to pay premiums from 2025 to 2035 will range from 10.7 to 16 thousand yuan.

**Conclusion:**

The finding shows that both the government and urban and rural residents can afford to pay future long-term care insurance costs; however, the long-term care insurance contributions of individual urban and rural residents in Nanning remain high. Hence, the Nanning municipal government should improve the awareness and participation rate of urban and rural residents in long-term care insurance; scientifically measure the financing level of long-term care insurance; establish a multichannel, scientifically reasonable, and dynamically adjusted long-term care insurance financing mechanism; and maintain the sustainable operation of the financing mechanism of long-term care insurance in Nanning.

## 1 Introduction

With the accelerating pace of global population aging, long-term care risk has emerged as a novel societal challenge beyond older adult poverty. Many individuals require prolonged medical care or personal assistance due to disabilities, significantly increasing fiscal pressures on governments and exacerbating the burden on pension systems (1). Long-term care insurance (LTCI) addresses this by pooling resources through collective solidarity and mutual aid, providing financial or service-based support for basic daily-living assistance and related medical care for individuals with chronic disabilities. This fundamentally enhances overall social welfare (2). Consequently, many nations are reshaping elder care systems and strengthening family support policies by establishing tailored long-term care service frameworks and complementary safeguards. For example, Germany, the first country to adopt a social insurance model for LTCI financing, implemented a broad-coverage, inclusive-access system in 1995. This system prioritizes meeting beneficiaries’ care needs, substantially improving the quality of life of populations with disabilities (3). Japan, the world’s most aged society, designed its LTCI system with a multi-stakeholder cost-sharing model (employees, employers, and government fiscal guarantees). By reducing reliance on government and societal subsidies, Japan has ensured greater sustainability in LTCI financing mechanisms (4). Drawing upon the advanced models in countries such as Germany and Japan, China has also progressively modernized its social security system since the launch its reform and opening-up in 1978.

According to the Fifth National Population Census, China entered an era of population aging in 2000, but its traditional reliance on family-based informal care has proven insufficient to meet the needs of populations with disabilities and their households (5). The Chinese government has prioritized addressing these challenges by integrating international best practices in LTCI and establishing a socialized LTCI system tailored to China’s shifting demographics and growing care demands. In 2016, China’s Ministry of Human Resources and Social Security issued the Guidelines for Piloting a Long-Term Care Insurance System, formally launching LTCI trials in 15 cities. By 2020, the pilot program expanded to 49 cities. Nanning City in the Guangxi Zhuang Autonomous Region—a less developed area in western China—was included in the second batch of pilot cities. Leveraging social insurance mechanisms and professional care services, Nanning has provided dual financial and care support to families of individuals with chronic disabilities, achieving tangible policy outcomes at this stage. As of May 2024, the program has benefited approximately 14,700 individuals with severe disabilities, reducing annual out-of-pocket costs by over 20,000 yuan per capita. However, Nanning’s current LTCI coverage is limited to urban employee medical insurance enrollees and excludes urban and rural residents. This undermines the inclusivity and equity of social security policies. If expanded to cover all residents, the financing mechanisms of LTCI must be sustainable in order to ensure its stable operation and high-quality development. Specifically, the LTCI fund must maintain a balanced revenue-expenditure structure (6).

A literature search has revealed several domestic and foreign studies that have achieved rich research results regarding LTCI financing from both macro and micro perspectives. From the perspective of research location, since the LTCI system was established earlier in high-income countries such as Germany and Japan, most domestic and foreign studies on LTCI financing have been conducted from more economically developed countries and regions in eastern China. For example, Doty et al. (7) analyzed the differences between the LTCI financing models of the U.S. and France in terms of policy design, premium cost, and coverage. Zhang et al. (8) measured the equilibrium of the LTCI fund in Xiamen City, China under different programs by simulating transfer indicators. Fewer studies have been conducted in less developed countries or regions, where the phenomenon of empty nesters is more prevalent. In these regions, the allocation of health resources is not reasonable, and it is more difficult to effectively solve issues pertaining to the treatment of illnesses and daily care faced by older adults (9).

In terms of research object, among China’s 49 LTCI pilot cities, the health insurance fund payment and individual contribution abilities of 29 (59.18%) cities do not cover residents due to varying economic development levels. Therefore, Chinese scholars have mostly examined LTCI financing for urban workers. For example, based on the theory of actuarial equilibrium of health insurance, Tao et al. (10) simulated the impact of the LTCI pilot expansion on the operation of Chinese urban workers’ health insurance fund by constructing a dynamic actuarial model. However, the development of China’s LTCI system is in its infancy, and the demand for LTCI for urban and rural residents, who make up the majority of the population, should not be ignored.

In terms of research methodology, population size and financing level are important variables in determining the LTCI financing mechanism. Several population forecasting methods are commonly used internationally, such as regression analyses and the gray model forecasting method; however, these models are limited to small measurement areas and shorter time periods. The regression analysis method, in particular, results in large errors when predicting the population for a longer period of time (11). By contrast, the PADIS-INT model has strong robustness and adaptability, as well as high measurement accuracy, and can be used to measure population size over a long period. Internationally, the Mannion method and multi-state Markov model are usually used to measure the level of LTCI financing. However, in practice, the Mannion method is unable to make long-term projections and the use of multi-state Markov models necessitates a large database of long-term care statistics, which is more complex to calculate and not as easy to realize (12). The ILO financing model, on the other hand, is based on the principle of break-even and can measure the level of insurance financing for a certain period. It is commonly used in the field of social health insurance with fewer constraints on the measurement.

In terms of research content, current domestic and international studies on LTCI financing mainly focus on drawing on the experience of typical countries’ financing mechanisms and using actuarial methods to predict the future contribution level of LTCI. Relatively few studies have analyzed and compared whether the sharing capacity of the main financing bodies is within a reasonable range and sustainable. In addition, most studies on the calculation of LTCI financing levels have ignored the impact of factors such as future population size and life expectancy growth, which may impact the accuracy of the calculation of future demand for LTCI services and financing pressures (6). This underestimates the level of financing required to realize the sustainable operation of the LTCI fund.

This study takes Nanning, China as an example. It uses the PADIS-INT and ILO financing models to measure the level of residents’ LTCI financing and the sharing capacity of the main financing bodies according to the actual situation in China and the principle of long-term actuarial equilibrium. In doing so, this study aimed to provide decision-making references for the improvement of China’s LTCI financing mechanism. Compared with existing research, this study verifies the possibility of realizing the sustainable operation of residents’ LTCI fund in less developed regions from multiple angles. First, it considers the impact of demographic factors, such as the extension of per capita life expectancy and urban–rural migration rate, when measuring the future size of the population with disability. This is conducive to accurately predicting the trend of China’s future population and the size of the population with disability in the insured group. Second, it combines the sharing ratios of the main LTCI contributors in different countries or regions, and analyzes the contribution burden and sustainability of different financing subjects in less developed regions.

## 2 Materials and methods

### 2.1 Data sources

The data used in this study were obtained from the following sources: Nanning’s population in 2020 from the Nanning Seventh Population Census Bulletin; Nanning’s life expectancy per capita in 2020 and 2030 from the Nanning Population Development Plan (2021–2030); Nanning’s fiscal revenue in 2021 from the Nanning Statistical Yearbook 2022; Nanning’s comprehensive basic medical insurance participation rate in 2021, 2023 urban workers’ LTCI participation rate, contribution rate, care treatment, management cost, and institutional home care selection rate from Nanning Municipal Healthcare Security Bureau; institutional home care service treatment standards from Nanning Long-Term Care Insurance System Pilot Implementation Opinions; Guangxi’s 2014–2024 economic growth rate from the National Economic and Social Development Statistics Bulletin of the Guangxi Zhuang Autonomous Region, 2015–2023 and the Communiqué of the Fifth National Economic Census of Guangxi; disposable income and consumer spending per capita of residents in 2014–2024 from the Guangxi Statistical Yearbook of 2015–2023, Statistical Communiqué of National Economic and Social Development of Guangxi Zhuang Autonomous Region of 2023, and Main Data on Income and Expenditures of Urban and Rural Residents of Guangxi of 2024; the disability rate refers to the predicted results of the future disability rate of China’s older adult population by Wang et al. (13); and the 2021 rolling balance of the Nanning Urban and Rural Residents Basic Medical Insurance Fund was obtained from the Nanning Urban and Rural Residents Basic Medical Insurance Transfer Payment 2021 Performance Self-Assessment Report.

### 2.2 Research methods

#### 2.2.1 Population projection based on the PADIS-INT model

PADIS-INT 1.0 is a population projection model independently developed by the China Population and Development Research Centre with the support of the United Nations Population Division. By introducing techniques such as non-linear projection models and multi-region dynamic equilibrium projections, it uses the gender-age structure of the starting population in the single-year age group as the input term for relevant projections and dynamically adjusts the parameters over the projection time to accurately predict future population development. It is suitable for population planning and applied forecasting in China, which has a large population size and uneven age structure (14).

#### 2.2.2 Forecasting funding levels based on the ILO model

The ILO financing model was proposed by the International Labour Organization and International Institute for Security Studies in 2000 and aims to follow the principle of overall balance of funds to measure the proportion of social health insurance financing in a health financing model (15). LTCI is a quasi-public good designed to provide social welfare and is part of social health insurance. Therefore, in this study, the ILO financing model was selected for the relevant parameter settings to simulate and measure the future contributions of LTCI for urban and rural residents.

#### 2.2.3 Testing the feasibility of equilibrium rate sharing based on EViews software

EViews is a tool for collecting information, and estimating, testing, designing, and applying models for forecasting and solving econometric research, with functions such as data processing, graphing, statistical analysis, modeling analysis, forecasting, and simulation. Using EViews software to analyze, model, and forecast per capita disposable income and consumer spending of urban and rural residents has strong operability and applicability (16). Therefore, this study uses EViews software to construct the fitting equations of the disposable income and consumer spending of urban and rural residents in Nanning over the year, predict changes in their disposable income and consumer spending from 2025 to 2035, and analyze the feasibility of balanced rate-sharing.

## 3 Results

### 3.1 Population size measurement

The population size measurement is based on the total population data of Nanning in 2020 from the Seventh Population Census Bulletin of Nanning City, and is measured using the PADIS-INT model. Section 3.1.1 explains the measurement of the PADIS-INT population projection model, considering the project parameters, migration level, population life expectancy, and life table.

#### 3.1.1 Parameter setting

##### 3.1.1.1 Project setting

Based on data from the seventh population census of Nanning in 2020, this study sets the starting year of the PADIS-INT model to 2020, the ending year to 2035, and the interval of parameter adjustment to five years to predict and analyze the trend of the age structure of the population in Nanning from 2023 to 2035.

##### 3.1.1.2 Migration level

Within the regional scope of Nanning, the factors that change the population size are mainly natural changes in the internal population and migration changes in the external population. However, according to demographic theory, when the size of the total population reaches the size of a provincial capital city, such as Nanning, its internal population age and gender structure tend to remain relatively stable (17). Therefore, this study does not consider the effects of migrating populations for the time being.

##### 3.1.1.3 Life expectancy *per capita*

Life expectancy is generally believed to be closely related to the level of medical care and socioeconomic development. Furthermore, continuous improvement in regional economic and social development can greatly increase life expectancy. The Nanning Population Development Plan (2021–2030) mentions that life expectancy in Nanning will be 77.7 years in 2020 and 79.6 years in 2030, which shows that life expectancy in Nanning will increase by about 1 year every 5 years on average. However, China has entered a new stage of life expectancy development since 2005, and the growth of life expectancy will slow down year by year (18). Therefore, this study assumes that the life expectancy per capita in Nanning will be 80 years by 2035.

##### 3.1.1.4 Life table

The PADIS-INT population projection model contains various life tables, such as the United Nations, Coale–Demeny, and Elandt–Johnson model life tables. Among these, the Coale–Demeny model is based on the largest number of actual life tables and is the most accurate. Therefore, this study adopts the Coale–Demeny model life table to predict the risk of population deaths in Nanning.

#### 3.1.2 Measurement results

According to the results of the PADIS-INT population projection model, the total population of Nanning shows a continuous growth trend from 8,741,600 people in 2020 to 9,108,400, 9,459,100, and 9,850,000 people in 2025, 2030, and 2035, respectively. The number of older adults aged 65 years old and above will grow from 931,900 (10.66%) in 2020 to 1,106,000 (12.14%), 1,411,100 (14.81%), and 1,715,600 (17.50%) in 2025, 2030, and 2035, respectively. These trends suggest that population aging in Nanning is becoming more serious. LTCI is an insurance benefit that mainly targets people with disabilities. It can be obtained when they need to continue receiving medical care at home or in a nursing facility due to old age, chronic diseases, or a state of disability (19). The size of the incapacitated population depends on two factors: the total population and level of disability.

##### 3.1.2.1 Total population

According to the statistical data of the Nanning Medical Security Bureau, the comprehensive participation rate of basic medical insurance calculated on the basis of the household population in Nanning was 95.24% in 2021. This study assumes that the ratio of the number of urban and rural residents’ basic medical insurance participants to the total population of Nanning in the period 2022–2035 is the same as that of the relevant ratio in 2021. Combined with the results in Table 1, the number of urban and rural residents’ basic medical insurance participants in Nanning can be calculated (see Table 1).

**Table 1:** Number of urban and rural residents enrolled in basic medical insurance and number of people with severe disabilities in Nanning City.

Year	Number of Participants (10,000 people)	Number of severely disabled (people)
2025	606.99	28,785
2030	630.35	35,226
2035	653.41	41,928

##### 3.1.2.2 Level of disability

At present, the population employing LTCI treatment in Nanning includes people with severe disabilities who meet the assessment criteria; therefore, this study focuses on the severity levels of disability. Data on the base of Nanning’s population that has disabilities are lacking. Therefore, this study refers to the medium scenario (2.82, 2.91, and 2.96% in 2025, 2030, and 2035, respectively) in the prediction results of Wang et al. (13) on the future disability rate of China’s aged population in lieu of the future severe disability rate of Nanning’s urban and rural population aged ≥65 years. Since the official implementation of the LTCI system in Nanning in 2021, the period from 2021 to 2022 was a trial phase, during which policies were continuously adjusted, resulting in relatively lower accuracy and completeness of statistical data. Therefore, this paper utilizes the long-term care insurance data from 2022 to 2023 in Nanning for calculations. According to statistics, the number of persons with severe disabilities aged under 65 years actually undergoing treatment under LTCI in Nanning between 2022 and 2023 was 1,360, and the number of urban workers aged under 65 years participating in the insurance in 2023 is 1,397,200. Based on this, the severe disability rate of the population of urban workers aged under 65 years is about 0.1%. Some urban workers with severe disabilities may not use LTCI and the population with severe disabilities among the urban and rural basic medical insurance participants may be slightly higher than that of the workers. Therefore, this study assumes that the severe disability rate of urban and rural residents aged under 65 in Nanning is 0.15%, and the severe disability rate of urban and rural residents aged under 65 will remain unchanged in the future. Based on the above assumptions, combined with the corresponding calculations in Table 1, the numbers of urban and rural residents who have severe disabilities in Nanning in 2025, 2030, and 2035 can be obtained (see Table 1).

### 3.2 Funding level measurement

This study adopts the ILO financing model, based on the principle of pay-as-you-go fund balance, to determine the fund revenue scale by measuring the scale of Nanning LTCI Fund expenditure from 2025 to 2035, and determines the financing level. The ILO financing model is mainly composed of sub-models such as the population estimation model, cost estimation model, and financing ratio.

#### 3.2.1 Demographic model

A demographic model was used to measure the size of the population participating in LTCI. Since LTCI is a quasi-public good and compulsory for the state to participate in, this study assumes that all urban and rural residents with basic medical insurance participate in LTCI, with a contribution rate of 100%. $LF\left(t\right)$ represents the population size covered by LTCI in year *t*, and is the number of urban and rural residents covered by basic medical insurance. The specific model is as follows:


$$LF\left(t\right)=\mathrm{POPACT}\left(t\right)$$

In this study, according to the calculation model and prediction results of the number of basic medical insurance participants for urban and rural residents in Table 1, the number of LTCI participants $LF\left(t\right)$ among Nanning residents in 2025, 2030, and 2035 is set at 606.99, 630.35, and 653.41 million, respectively.

#### 3.2.2 Cost estimation model

The cost estimation model primarily includes nursing, management, and other expenses.


$$TE\left(t\right)=\mathrm{BE}\left(t\right)+AE\left(t\right)+OE\left(t\right)$$

where $TE\left(t\right)$ is the total cost of the older adult care insurance plan, $\mathrm{BE}\left(t\right)$ represents the first year *t*’s older adult care insurance fees, $AE\left(t\right)$ represents the first year *t*’s older nursing risk management fees, and $OE\left(t\right)$ represents the first year *t*’s older adult care insurance cost to weed out the nursing and management costs of other spending.

In the actual operation of the LTCI for employees in Nanning, the monthly fixed standard for nursing treatment is determined at 50% of the weighted average monthly salary of full-caliber personnel in urban non-private units and urban private units in the Guangxi Zhuang Autonomous Region for the year 2019 (i.e., RMB 2,463). The development of the integration of China’s health insurance system has amply demonstrated that differences between urban and rural areas, geographic regions, groups, and ages undermine the fairness of the system and tend to lead to system fragmentation (20). In order to fully reflect the fairness of the LTC insurance system, this study therefore sets the LTCI care treatment for Nanning residents in line with the treatment standards for employees. That is, the flat rate of RMB 2,463 per person per month (RMB 29,556 per year) is used as the base of the care cost expenditures for calculating the care cost expenditures for the years 2025, 2030, and 2035.

**Figure 1:**
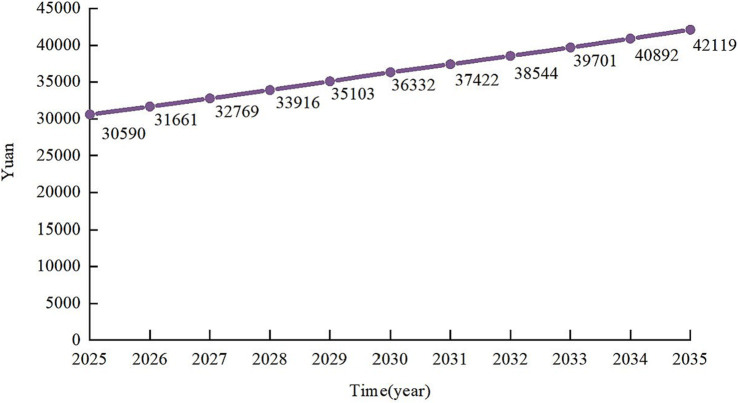
Forecast of long-term care insurance care benefits for urban and rural residents in Nanning, 2025–2035.

According to data from the National Economic and Social Development Statistics Bulletin of the Guangxi Zhuang Autonomous Region, Guangxi’s economic growth rate has continued to slow down in recent years, with economic growth rates of 8.3, 7.9, 7.0, 7.1, 6.8, 6.0, 3.7, 7.5, 2.9, 4.1, and 4.2% from 2014 to 2024, respectively. This phenomenon is also consistent with the common law of economic growth in the world. That is, after the per capita GDP of a country exceeds 10,000 U.S. dollars, it steps into the ranks of middle-income countries. Its economic growth will enter a stage of stabilized growth mainly to improve quality, and the growth rate will generally slow down (21). For this reason, this study, based on the law of economic growth and actual situation of Guangxi’s economic development and in accordance with the principle of positive and steady, leaving room for maneuver, assumes that the average annual growth rate of Nanning’s economic development level is 3.5% from 2025 to 2030, and 3.0% from 2031 to 2035, and that the growth rate of the LTC nursing treatment grows at the same rate as the growth rate of the economic development level, which can be calculated as the level of LTCI nursing treatment for urban and rural residents in Nanning will grow from 30,590 yuan to 42,119 yuan (see Figure 1).

Operational data during the pilot period of LTCI in Nanning show that, as of March 2023, as many as 90.67% insured persons actually using LTCI services chose institutional home care services. According to the Nanning Long-Term Care Insurance System Pilot Implementation Opinions and other documents, those who choose institutional in-home care services will be paid 75% of the monthly care treatment standard by the LTCI fund, and the remaining 25% will be paid by individuals. Therefore, this study assumes that all LTCI participants in Nanning will choose to receive at-home care services from designated care service agencies. After excluding 25% of LTCI costs to be paid by individuals, the total financial demand for LTCI for urban and rural residents in Nanning can be obtained (see Table 2).

**Table 2:** Cost of care, total financial need, and population financing levels for urban and rural residents of Nanning City’s long-term care insurance in 2025, 2030, and 2035 (yuan).

Year	Care costs (yuan)	Total financial demand (10,000 yuan)	Financing levels (10,000 yuan)
2025	88054.64	66040.97	108.80
2030	127982.26	95986.98	152.28
2035	176594.85	132446.13	202.71

#### 3.2.3 Funding ratio model

Based on the above calculations and the established demographic and cost estimation models, and in accordance with the principle of the pay-as-you-go system, “expenditure determines revenue,” the amount of financing for LTCI for urban and rural residents can be calculated by substituting the results into the model.


$$\mathrm{CONT}\left(t\right)=LF\left(t\right)$$

$$\mathrm{PAYGR}\left(t\right)=TE\left(t\right)/\mathrm{CONT}\left(t\right)$$

$\mathrm{CONTi}\left(t\right)$ denotes the number of contributors of category i people in the first year. $\mathrm{PAYGR}\left(t\right)$ indicates the amount of funding for long-term care insurance for urban and rural residents in the year t . The annual contribution amount of urban and rural residents in Nanning increases from 108.80 yuan in 2025 to 202.71 yuan in 2035, with an average annual growth rate of 6.34% (see Table 2).

### 3.3 Feasibility analysis of equalized rate sharing

The sustainability of LTCI fund operations depends on the complementary roles of multiple main bodies. At present, the international, more mature LTCI implementation of national financing sources includes government financial subsidies, user units, and individual contributions, while the rate-sharing ratio is based on the distribution of economic development over various places, users, governments, enterprises, and individuals. Therefore, regarding the sharing ratio of financing responsibility, this study refers to Japan, which has also built a social LTCI system that is similar to China’s national conditions and distributes the financing responsibility of LTCI evenly, with the government and individuals each bearing 50% of the financing responsibility of LTCI (22).

#### 3.3.1 Feasibility analysis of government sharing

The feasibility of government sharing can be tested using the ratio of the total fiscal expenditure to regional fiscal revenue. However, since the Nanning municipal government has ceased to disclose its fiscal revenue starting from 2022, this study takes the fiscal revenue of 82.884 billion yuan in 2021 published in Nanning Statistical Yearbook 2022 as the base, and measures it according to the principle that the economic growth rate will be 2.9, 4.1, and 4.2% in 2022–2024, 3.5% in 2025–2030, and 3.0% in 2031–2035. The results show that the fiscal revenue of Nanning will increase from 82.884 billion yuan in 2021 to 95.751, 113.723, and 131.836 billion yuan in 2025, 2030, and 2035, respectively. According to the 50% sharing of LTCI financing responsibility between the government and individuals, the Nanning municipal government’s financial responsibility for LTCI costs for urban and rural residents will increase from 330 million yuan in 2025 to 662 million yuan in 2035, accounting for a relatively small proportion of the current year’s fiscal revenue, between 0.34 and 0.50% (see Table 3).

**Table 3:** Nanning long-term care insurance system rate sharing—the government and individuals each bearing 50% of the financing responsibility of LTCI.

Year	Urban and rural residents in payment amount (yuan/person)	Personal expenses (yuan/person)	Costs borne by the government (yuan/person)	Number of urban and rural residents enrolled in LTCI (million people)	Government expenditure (billion yuan)	The proportion of government expenditure in the fiscal revenue of the year(%)
2025	108.80	54.40	54.40	606.99	3.30	0.34
2030	152.28	76.14	76.14	630.35	4.80	0.42
2035	202.71	101.35	101.35	653.41	6.62	0.50

#### 3.3.2 Feasibility of residential sharing

The ability of individual residents to bear contributions is closely related to their disposable income; the higher their disposable income, the higher their ability to pay. Since Nanning lacks relevant data on the per capita disposable income and consumer spending of residents in some years, and the per capita disposable income and consumer spending of Nanning residents are higher than that of the average level of the entire region of Guangxi. Therefore, this study utilizes Eviews to calculate the per capita disposable income and consumption expenditure data of Guangxi residents, estimates the parameter values of the income-expenditure model, and constructs a fitting equation of income and consumption expenditure against the year, specifically a unitary linear regression equation, to verify the individual payment capacity of Nanning residents for long-term care insurance from 2025 to 2035.

**Figure 2:**
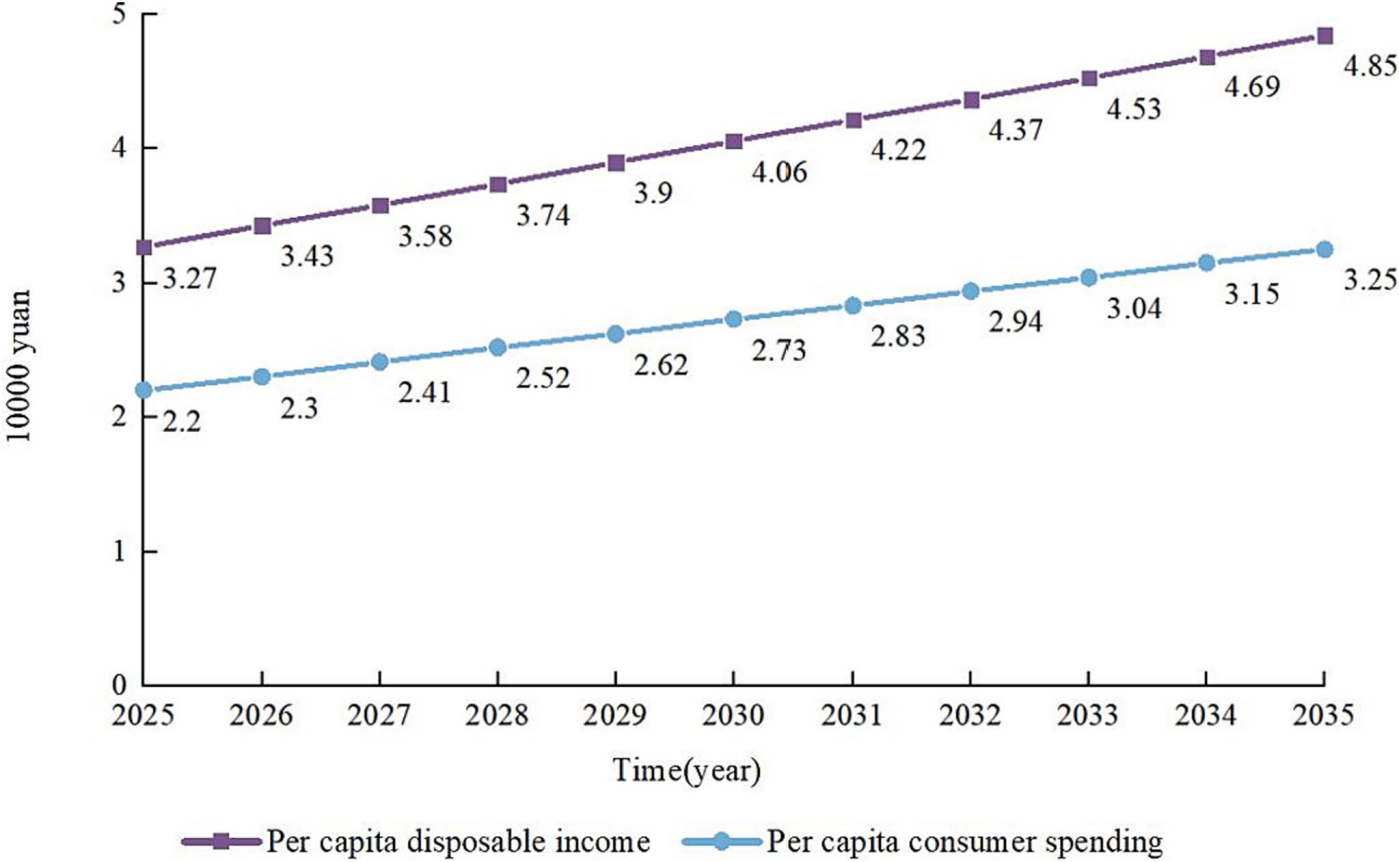
Disposable income and consumer spending per capita in Guangxi, 2025–2035.

By processing the data with Eviews, it is found that there is a linear relationship between the per capita disposable income and consumption expenditure of Guangxi residents from 2014 to 2024. Unary linear fitting was applied to the data, and the fitting results showed that $R^{2}$ was 0.999 and 0.990, which are close to 1, indicating that the degree of fit was very high. The unitary linear regression model of per capita disposable income and consumer spending of Guangxi residents can be obtained as follows. Substituting the years into the unitary linear regression model yields the per capita disposable income and consumer spending of Guangxi residents from 2025 to 2035 (see Figure 2).


Y=0.157909X−316.4966

Y=0.105545X−211.5299

Income is the primary determinant of the level of consumption; the greater the disposable income of the population, the greater its ability to purchase goods and services, and the greater its corresponding consumption, excluding other conditions. Keynes proposed that income consists of consumption, investment, and other components, and consumption and income are in a functional relationship that can be expressed by the linear function C=a+bY . To simplify the calculation process, in this study, when measuring the maximum contribution capacity of residents, it is assumed that their income is only used for current consumption and savings; that is, the maximum amount of insurance premiums borne by residents in the current period is their current savings. In addition to consumption, income can be used to pay insurance premiums, so the maximum rate of contribution that can be afforded by residents is as follows:


$$B/Y=\left(Y-C\right)/Y=1-b-a/Y$$

where *C* is consumption, *Y* is disposable income, *a* is spontaneous consumption, *b* is marginal propensity to consume, and *B* is savings. The formula shows that to calculate the maximum contribution rate, the functional relationship between disposable income and consumption must first be determined. Therefore, this study used EViews software to simulate the disposable income and consumer spending trends.

For disposable income and consumer spending, a linear regression analysis was conducted. The results showed that $R^{2}=0.9910$ , the goodness of fit was high, the *T*-test and *F*-test were passed, and the regression equation was obtained:


C=0.668389Y+0.014485

The equation shows that for every 10,000 yuan increase in income, residents can use about 6.7 thousand yuan for consumption and about 31% for savings, that is, the amount of money that Guangxi residents can use to pay insurance premiums in 2025–2035 will be 10,700–16,000 yuan. With reference to the projected value of the maximum affordability level of individual premium payments by Guangxi residents from 2021 to 2035, a large gap still exists between the cost borne by individuals for LTCI mentioned in the Table 2 and the maximum affordability of individual premium payments in the future. That is, residents in Nanning have income available for LTCI.

### 3.4 Sensitivity analysis

In order to test the stability of the measurement results and examine whether the research conclusions will be biased due to changes in certain parameters, under the premise of retaining the basic assumptions, this study conducts a sensitivity test on the parameters of the proportion of financing responsibility sharing and the reimbursement ratio of nursing treatment.

#### 3.4.1 Sensitivity analysis of financing responsibility sharing ratio

Considering the affordability of individuals, governments and organizations, determining a reasonable rate sharing ratio of LTCI is directly related to the smooth operation of its financing. Therefore, this study starts from objective indicators in all the pilot cities of LTCI in China, such as the number of resident population, number of urban workers’ basic medical insurance participants, number of urban and rural residents’ basic medical insurance participants, and per capita disposable income. This follows the division of LTCI responsibility that is done in Changchun City, which has a similar overall development situation to Nanning. The ratio of individual contributions to financial subsidies is 5:1 for the calculation, that is, 83.33% of the total cost of the Changchun LTCI is borne by individuals in Nanning, and 16.67% is borne by the Nanning Municipal Government. According to Table 4, the proportion of the urban and rural residents’ LTCI costs borne by the Nanning Municipal Government to the current year’s fiscal revenue is between 0.11 and 0.17%, which is relatively small, and the costs borne by the individual residents are also within the maximum affordability of the Guangxi residents’ future individual insurance premiums (10,700–16,000 RMB). Therefore, adjusting the proportion of responsibility for financing will not change the basic conclusions of the section 3.3.2.

**Table 4:** Nanning long-term care insurance system rate sharing—83.33% of the total cost of the LTCI is borne by individuals, and 16.67% is borne by the government.

Year	Urban and rural residents in payment amount (yuan/person)	Personal expenses (yuan/person)	Costs borne by the government (yuan/person)	Number of urban and rural residents enrolled in LTCI (million people)	Government expenditure (billion yuan)	The proportion of government expenditure in the fiscal revenue of the year(%)
2025	108.80	90.66	18.14	606.99	1.10	0.11
2030	152.28	126.89	25.38	630.35	1.60	0.14
2035	202.71	168.91	33.79	653.41	2.21	0.17

#### 3.4.2 Sensitivity analysis of reimbursement rate of nursing treatment

In addition to the financing level, the reimbursement ratio of nursing treatment is also a key factor affecting the continuous payment of the LTCI fund and the development of the system continuity. According to the Nanning Long-Term Care Insurance System Pilot Implementation Opinion and other documents, the highest reimbursed nursing service mode of Nanning LTCI is the institutional in-home nursing care, with a fund payment ratio of 75%, while the fund payment ratios for institutionalized nursing care and off-site residential nursing care are 70 and 60%, respectively, which is lower than that of Shanghai, Qingdao, and other economically more developed cities in China, as well as that of South Korea (23) and other economically developed countries. Therefore, if the treatment standard of Nanning LTCI participants who choose institutional home care services increases by 5% on the original basis for measurement, the Nanning government will use 0.37–0.54% of its fiscal revenue for the year to bear the cost of LTCI for urban and rural residents, which is still a small proportion. The cost borne by individual residents is also within Guangxi residents’ maximum affordability to pay for insurance premiums individually in the future (10,700–16,000 RMB). This indicates that adjusting the reimbursement ratio for nursing treatment will not change the basic conclusions of the previous article (see Table 5).

**Table 5:** Nanning long-term care insurance system rate sharing—the reimbursement rate for nursing care benefits is 80%.

Year	Urban and rural residents in payment amount (yuan/person)	Personal expenses (yuan/person)	Costs borne by the government (yuan/person)	Number of urban and rural residents enrolled in LTCI (million people)	Government expenditure (billion yuan)	The proportion of government expenditure in the fiscal revenue of the year(%)
2025	116.05	58.03	58.03	606.99	3.52	0.37
2030	162.43	81.21	81.21	630.35	5.12	0.45
2035	216.21	108.11	108.11	653.41	7.06	0.54

## 4 Discussion and recommendations

As the population continues to age, the following should be prioritized to facilitate the sustainable operation of the LTCI fund: First, the supporting capacity of the fund must be improved in order to cater to the large number of residents it covers. Second, the extent of individual residents’ and the government’s responsibility in financing the LTCI, as well as their capacity to bear it, should be reasonably determined. Based on the principle of balance of payments and revenues, this study measures the financing level and sharing capacity of LTCI for Nanning residents through PADIS-INT and ILO financing models. The results show that the cost of LTCI is within the affordable range of Nanning residents and the government.

The government is currently not responsible for providing funds in the financing process of Nanning’s employee LTCI. Only in the subsequent distribution process does it carry out a macro-control of the LTCI fund to ensure that the funds are sufficiently and smoothly distributed (24). This may be due to Nanning, which is an underdeveloped region in western China, having entered a period of low-speed economic growth since 2020, limiting the government’s financial resources. However, theoretically speaking, the LTCI system was provided by the government as an important component of China’s social security system to help realize a fair distribution of social wealth, reduce the burden of long-term care for the disabled, and prevent the polarization of wealth under the conditions of the market economy. In fact, the proportion of the urban and rural residents’ LTCI expenses borne by the Nanning municipal government are lower than 0.6%, which is less than the proportion of financial funds contributed by the government in the LTCI system in more economically developed countries such as Germany (0.9%), Japan (1.4%) (25), and China (1.62–2.07%) (26). In a word, in the process of system operation to provide institutional protection for the LTCI’s financing mechanism, the government does not only act as the main body in charge of policy formulation, but also as the gatekeeper for LTCI financing. It should optimize the articulation between various policies to provide certain financial subsidies for the normal operation of the LTCI system (27). Moreover, it should actively expand third-party fund sources of income, such as private donations and the Fortune Lottery Public Welfare Funds, to alleviate the financial pressure on residents, thereby guaranteeing the sustainable operation of the LTCI fund.

According to the 50% split in LTCI financing responsibility between the government and individuals, the amount of individual contribution for urban and rural residents’ LTCI in Nanning will increase from RMB 54.40 in 2025 to RMB 101.35 in 2035. This is closer to the findings of Ma (28) regarding the LTCI contribution of residents in Anhui Province (96.77 RMB); however, Soga (29), Sano (30), and other scholars have found that increasing the individual out-of-pocket payment will reduce the rate of demand and utilization of LTCI services. Amid the current economic downturn, the rising individual LTCI contribution may bring a certain degree of financial burden to Nanning residents. This may trigger a backlash of public opinion and lower residents’ willingness to participate in the insurance program. Therefore, when establishing a financing mechanism for residents’ LTCI in the future, the Nanning government should strengthen the publicity of LTCI in grass-roots communities, and ensure that the LTCI policy can be implemented through the continuous creation and promotion of the “Yong Xiao Hu” featured service brand and publicity column. In doing so, LTCI can be better integrated into the daily lives of the residents (31). In addition, flexible subsidy standards for LTCI should be set in light of the actual situation of Nanning residents. The LTCI funding level and individual contributions must be dynamically adjusted on a regular basis in accordance with factors such as population aging, disability rates, and levels of socio-economic development and considering the continuation and organic fusion of the LTCI fund with the old-age subsidy, subsidy for persons with severe disabilities, and other social welfare funds. According to the Nanning Urban and Rural Residents’ Basic Medical Insurance Transfer Payment 2021 Performance Self-Assessment Report, the rolling balance of the Nanning Urban and Rural Residents’ Basic Medical Insurance Fund in 2021 was RMB 4.266 billion, which is relatively large. In addition to the direct input from the financial sector, the government may also consider allocating a portion of the Nanning Urban and Rural Residents’ Basic Medical Insurance Fund to alleviate the pressure on the funding of the residents’ LTCI and promote the flow of funds. On the premise of the smooth operation of other insurance systems, the government should also adjust the contribution structure of other insurance types. Where income exceeds expenditure, it should moderately reduce the contribution rate of such types of insurance in order to make room for LTCI contributions.

In the current context of low fertility, large-scale population migration between urban and rural areas, as well as the weakening of the family care function, managing the care of older adults with disabilities amid an aging population is a vital issue. Therefore, this study suggests that the main body for LTCI financing in Nanning draw insights from the sustainable development of LTCI in Germany and South Korea. The principle of “care insurance follows medical insurance” should be followed, so that all medically insured individuals over 18 years old can have access to LTCI. The responsibility mechanism of the reciprocal rights and obligations of individuals, enterprises, and the government in the financing process should also be clarified (32). Pay-as-you-go LTCI financing and an independent financing mechanism can be implemented to maintain the mutual independence of the financing channels of LTCI and medical insurance, determine the level of financing, and fairly divide the financing responsibilities of all parties, thereby guaranteeing the sustainable operation of the LTCI financing mechanism. Further, in-depth field surveys can be conducted to introduce policies and measures that are in line with the level of Nanning’s economic development, old-age habits, and other actual circumstances, to effectively increase residents’ motivation to participate in the insurance program and enhance their sense of security. Residents can also play an active role by voicing their opinions about the LTCI policy through multiple channels, such as the Nanning Government-Citizen Interaction Information Platform and the official website of the Medical Protection Bureau.

This study has some limitations. First, the measurement results in this study are realized under a series of assumptions, which are carried out in an ideal state. In reality, other factors, such as the influence of policies and social environment, may cause the measurement results to deviate to a certain extent from the actual situation in Nanning in the future. Second, when considering the cost factors of LTCI, due to the large proportion of nursing costs and for the convenience of measurement, this study does not include the 5% cost of services required by the contracting organization in the calculation. Further adjustments and improvements for accuracy are necessary in future studies.

## Data Availability

The raw data supporting the conclusions of this article will be made available by the authors, without undue reservation.
